# The natural history of infantile neuroaxonal dystrophy

**DOI:** 10.1186/s13023-020-01355-2

**Published:** 2020-05-01

**Authors:** Fadie D. Altuame, Gretchen Foskett, Paldeep S. Atwal, Sarah Endemann, Mark Midei, Peter Milner, Mustafa A. Salih, Muddathir Hamad, Mohammad Al-Muhaizea, Mais Hashem, Fowzan S. Alkuraya

**Affiliations:** 1 College of Medicine, Alfaisal University, Riyadh, Saudi Arabia; 2 Retrotope Inc., Los Altos, CA USA; 3 Division of Pediatric Neurology, Department of Pediatrics, College of Medicine, King Saud University, Riyadh, Saudi Arabia; 4 Department of Neurosciences, King Faisal Specialist Hospital and Research Center, Riyadh, Saudi Arabia; 5 Department of Genetics, King Faisal Specialist Hospital and Research Center, Riyadh, Saudi Arabia

**Keywords:** Infantile neuroaxonal dystrophy, INAD, Natural history, Molecular genetics

## Abstract

**Background:**

Infantile neuroaxonal dystrophy (INAD) is a rapidly progressive neurodegenerative disorder of early onset causing premature death. It results from biallelic pathogenic variants in *PLA2G6*, which encodes a calcium-independent phospholipase A2.

**Objective:**

We aim to outline the natural history of INAD and provide a comprehensive description of its clinical, radiological, laboratory, and molecular findings.

**Materials and methods:**

We comprehensively analyzed the charts of 28 patients: 16 patients from Riyadh, Saudi Arabia, 8 patients from North and South America and 4 patients from Europe with a molecularly confirmed diagnosis of *PLA2G6*-associated neurodegeneration (PLAN) and a clinical history consistent with INAD.

**Results:**

In our cohort, speech impairment and loss of gross motor milestones were the earliest signs of the disease. As the disease progressed, loss of fine motor milestones and bulbar dysfunction were observed. Temporo-frontal function was among the last of the milestones to be lost. Appendicular spastic hypertonia, axial hypotonia, and hyperreflexia were common neurological findings. Other common clinical findings include nystagmus (60.7%), seizures (42.9%), gastrointestinal disease (42.9%), skeletal deformities (35.7%), and strabismus (28.6%). Cerebellar atrophy and elevations in serum AST and LDH levels were consistent features of INAD. There was a statistically significant difference when comparing patients with non-sense/truncating variants compared with missense/in-frame deletions in the time of initial concern (*p* = 0.04), initial loss of language (*p* = 0.001), initial loss of fine motor skills (*p* = 0.009), and initial loss of bulbar skills (*p* = 0.007).

**Conclusion:**

INAD is an ultra-rare neurodegenerative disorder that presents in early childhood, with a relentlessly progressive clinical course. Knowledge of the natural history of INAD may serve as a resource for healthcare providers to develop a targeted care plan and may facilitate the design of clinical trials to treat this disease.

## Introduction

Infantile neuroaxonal dystrophy (INAD, NBIA2A; MIM# 256600) is a major subtype of *PLA2G6*-associated neurodegeneration (PLAN), a heterogenous group of clinical disorders which additionally includes atypical neuroaxonal dystrophy (NBIA2B; MIM# 610217) and adult-onset dystonia-parkinsonism (PARK14; MIM# 612953). The diagnosis of PLAN is established by identifying biallelic pathogenic variants in *PLA2G6*, while determining the specific phenotype of PLAN is based on various clinical, neurophysiologic, radiographic, and laboratory features.

*PLA2G6*, or phospholipase A2 group VI gene, encodes an 85/88 kDa calcium-independent phospholipase A2 that belongs to the phospholipase A2 family and catalyzes the hydrolysis of the sn-2 fatty acyl bonds in phospholipids, generating lysophospholipids and free fatty acids [1]. It is located on chromosome 22 at 22q13.1 [2] and contains 17 exons which span more than 69 kb [3]. Pathogenic variants in *PLA2G6* lead to failed repair of oxidative damage to phospholipid membranes and result in adverse changes in membrane permeability and fluidity, a mechanism which may underlie the pathology of INAD [1, 3, 4]. The high metabolic demands and exposure to oxidative stressors typical of nervous system cells, particularly in some areas such as cerebellum and the basal ganglia [4, 5], make these structures highly sensitive to defects in phospholipase A2 activity.

The onset of INAD typically occurs between 6 months and 3 years of age, commonly presenting with psychomotor regression, gait disturbance, truncal hypotonia, and in some patients, strabismus and nystagmus. The disease progresses into spastic tetraparesis with symmetric pyramidal signs, progressive cognitive decline, optic atrophy, and bulbar dysfunction. The progression is usually rapid, and patients rarely survive beyond their first decade even with supportive care [5–8]. Seizures may present early or late in the disease course but are reported in only a minority of patients [5, 7, 9–11]. Histopathology may show the presence of axonal spheroid bodies in both the central and peripheral nervous system [5, 9, 12], and neuroimaging usually reveals cerebellar atrophy and, in some cases, iron accumulation in the globus pallidus.

Natural history of a disease is defined as the “natural course of a disease from the time immediately prior to its inception, progressing through its presymptomatic phase and different clinical stages to the point where it has ended and the patient is either cured, chronically disabled, or dead without external intervention” [13]. Defining the natural history of INAD is crucial to demonstrate its relentless and progressive nature and to identify markers of disease status and progression. Such information will be essential to plan any future interventional drug trials for INAD. Additionally, defining the natural history will help physicians develop appropriate and timely management and follow-up strategies and will inform patients and/or their surrogates of the prognosis of the disease.

The rarity of INAD has limited the ability to report on clinical observations in significant numbers of patients in any one study. This present retrospective international multicenter study seeks to present comprehensive clinical findings in the largest number of INAD patients consolidated in any single study to date. This international cohort of molecularly confirmed cases was collected and their clinical, radiographic, laboratory, and neurophysiologic features were documented to serve as a comprehensive resource for providers (and families) to consult in developing a targeted care plan, complete with education about potential complications and appropriate surveillance and support.

## Materials and methods

### Ethical approval

An IRB-approved written informed consent was obtained from all patients and their parents enrolled in this study. The ethics committee at King Faisal Specialist Hospital and Research Centre, Riyadh (KFSHRC) and Quorum Review (RAC# 2121053, File #: 33273/1; Protocol #: RT-INAD-NH001) specifically approved this study.

### History and physical examinations

The medical records of 28 patients in total, 16 from Saudi Arabia (P13 to P28), 8 from North and South America, and 4 from Europe, with molecularly and clinically confirmed diagnoses of INAD were analyzed comprehensively. The Saudi Arabian cohort patients were evaluated at KFSHRC and King Khalid University Hospital, College of Medicine, King Saud University, Riyadh, while the other cohort of patients were evaluated at multiple centers across North and South America and Europe. All patients underwent thorough neurological evaluations to document their clinical status at the time of their first visit and at each follow-up visit. A comprehensive chart review was done to extract relevant information, including birth history, family history of the disease, parental consanguinity, developmental history, age at disease presentation, disease progression, and neurological and ophthalmologic exam findings. All comorbid findings found in the records were reported. To ensure the patient histories were as accurate and complete as possible, patients’ families were interviewed to confirm and clarify information gained from the medical records when necessary.

### Neurophysiologic testing

EEG recordings were performed for 18 patients (64.3%). Electromyography (EMG), nerve conduction velocity (NCV), visual evoked potentials (VEP), and brainstem auditory evoked response (BAER) were also performed and recorded following a conventional protocol for some patients as a part of their clinical care. Additionally, one nerve biopsy and 4 muscle biopsies were taken and analyzed from four patients.

### Imaging studies

Magnetic resonance imaging (MRI) was performed on 26 patients (92.9%). No MRI results were found in the medical records of P2 and P9. MRI was repeated for 6 of those patients (23.1%). All MRI results were reviewed and confirmed by a neuroradiologist and pediatric neurologist.

### Laboratory tests

Aspartate aminotransferase (AST) and alanine aminotransferase (ALT) serum values were extracted from the records of 23 patients (82.1%). Some patients had various other laboratory tests performed, including complete blood count (CBC), lactate dehydrogenase (LDH), creatine kinase (CK), amino acid panel, renal function tests, ammonia levels, and lactate levels. All abnormal values were extracted from their medical records.

### Variant detection and classification

*PLA2G6* variants were identified in the patients using a multi-gene panel for neurological disorders or whole-exome sequencing as described previously [14–17]. Variants were classified according to American College of Medical Genetics & Genomics (ACMG) guidelines.

## Results

The phenotypes of individual patients are comprehensively outlined in supplemental Table S1.

### Birth history and family history

Nineteen (67.9%) patients were delivered at full term with no maternal or fetal complications. The mother of P20 was diabetic and had induction of labor at 38 weeks. After delivery, her neonate was admitted to the to the neonatal intensive care unit (NICU) for jaundice and respiratory distress. The mothers of P1 and P4 had pregnancies complicated by pre-eclampsia and immune thrombocytopenia, respectively. P4 was admitted to the NICU for thrombocytopenia. The mother of P3 had gestational hypertension and perception of decreased fetal movements in the 3rd trimester. P3 was admitted to the NICU for hypoxia and concern for streptococcus bacteremia. Two other patients (P13 and P14) were also admitted to the NICU for hypoxia. P5 delivery was complicated by fetal distress, not otherwise specified. The pregnancies of P12 and P16 were complicated by polyhydramnios. P12’s pregnancy was additionally affected by first trimester toxoplasmosis.

P1 and P2 are siblings from one family, and P15 and P16 are siblings from another family. P3, P17, P18, P21, P22, P23, P25, P26, and P27 have siblings or cousins identified as having a neurodegenerative disease or symptoms similar to those manifested in the affected patients but were not available for testing and confirmation. Overall, 15 patients (53.6%) came from consanguineous families.

### Developmental history and disease presentation

Nineteen patients (67.9%) had at least one delayed developmental milestone prior to the continued developmental regression that characteristically followed. The remaining nine patients reportedly had normal development prior to the regression.

Of the 19 patients reported to have developmental delay, 17 (89.5%) had some form of speech delay; four of these 17 were never able to speak. Sixteen of the 19 patients (84.2%) had some form of gross motor delay. Walking was the most commonly delayed gross motor milestone with 14 patients (87.5%) having some form of delay. Ten out of the 14 patients were never able to walk independently. Of the 16 patients with delayed gross motor milestones, six had a delay in sitting independently (37.5%), four in standing independently (25.0%), four in cruising (25.0%), three in rolling over (18.8%), and one in achieving head control (6.25%).

In addition to speech delay, P21 was reported to have gross and fine motor delay. P13 was reported to have delayed milestones, but no specifics were mentioned in his medical records.

Initial concerns arose when the parents noticed one or more delayed milestones (gross motor delay or a speech delay) or, more commonly, when they noticed developmental regression (with loss of balance being the most frequently cited). The average age at which initial concerns arose was 15 months (median = 14 months, range 6–36 months), with loss of balance being the most commonly reported initial concern (57.7%), followed by developmental delay (34.6%).

### Developmental regression and disease progression

Regardless of the presence or absence of developmental delay at an earlier age, all patients eventually experienced some form of developmental regression and lost some or all acquired developmental milestones at varying rates. Gross motor and speech milestones were the first milestones to be affected. Specifically, disturbance in balance (e.g. waddling gait, ataxic gait) was commonly reported by parents to be the first milestone affected (median = 14 months, mean = 16.5 months, range 9–36 months). In patients who never walked, losing the ability to stand (median = 21.5, mean = 25.1 months, range 11–31 months) and the ability to cruise (median = 21 months, mean = 21 months, range 18–24 months) were the most common first milestones to be lost. Overall, patients lost the ability to stand without support at a mean age of 25.1 months (range 11–50 months). Language was the next to be affected at a mean age of 27.6 months (median = 24.5 months, range 12–54 months).

As the disease progressed, patients started losing earlier gross motor milestones, such as crawling (median = 27 months, mean = 30.8 months, range 12–51 months), rolling over (median = 33 months, mean = 35.6 months, range 24–50 months), sitting unsupported (median = 33.5 months, mean = 35.9 months, range 24–57 months), and holding head upright (median = 34 months, mean = 53.7 months, range 34–94 months). Fine motor milestones were next to be affected, with a mean age of 47.8 months (median = 30 months, range 14–144 months). Finally, patients experience a gradual deterioration in their bulbar function, starting with choking on thin liquids or drooling (median age = 24 months, mean age = 27.8 months, range 22–39 months), progressing to inability to swallow solid food and being placed on a puréed diet (median age = 41 months, mean = 53.1 months, range = 18–132 months) and eventually requiring placement of a nasogastric tube (median = 6.5 years, mean = 6.9 years, range 2.6–12 years) or gastrostomy tube (median = 3.8 years, mean = 4.8 years, range 2.9–7.5 years). Responsiveness to verbal commands and interaction with parents or examiners were variably affected among patients and were reported to be one of the last milestones to be lost (median = 45.5 months, mean = 57.1 months, range 23 months to 15 years).

**Fig. 1:**
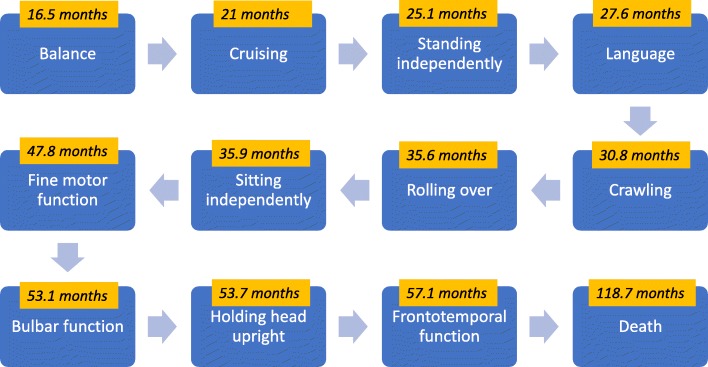
Timeline of the Natural History of INAD. Timeline graph representing the mean age (yellow box) at which each milestone (blue box) was lost

Six patients (21.4%) died during the course of the study at an average age of 9.9 years (median = 9.9 years, range 6–14.6 years). The causes of death for 2 patients were reported in their charts. P15 was brought to the ER after cardiac arrest at home, recovered normal cardiac rhythm after 14 min of CPR, then was intubated and ventilated. DNR (do not resuscitate) status was assigned and she died at the age of 10 years and 9 months (preliminary cause of death: cardiac arrest). P20 was admitted to PICU with respiratory distress (type 2 respiratory failure) that required intubation and ventilation. DNR status was assigned and she died at the age of 14 years and 7 months (preliminary cause of death: cardiopulmonary arrest). The cause of death of the remaining patients was solicited from the parents, the majority of whom reported a “respiratory issue” that preceded the patients’ deterioration and death (Fig. 1).

### Clinical examinations

Neurological examination revealed axial hypotonia in 12 patients (42.9%) and appendicular spastic hypertonia in 22 patients (78.6%). Eight of the 22 patients with appendicular spastic hypertonia had contractures in one or more joints. Generalized hypotonia and appendicular hypotonia were observed in four (14.3%) and two patients (7.1%), respectively. Seven patients (25.0%) had hyporeflexia on deep tendon reflex (DTR) examination, while 15 patients (53.6%) displayed hyperreflexia. Two patients (P26 and P10) had absent DTR, and one patient (P2) initially had hyperreflexia at 30 months of age followed by hyporeflexia at 35 months. P9 had hyporeflexia limited to the lower limb, while P17 had hyperreflexia limited to the lower limb. Two patients (P18 and P9) had hyperreflexia limited to the upper limb. Four patients (14.3%) were reported to have “ataxia” on physical examination records, but there were 17 patients with loss of balance that may have been the manifestation of undocumented truncal or appendicular ataxia. Similarly, there were also 14 patients with inability to sit independently and 7 with inability to stand independently that may have been manifesting truncal ataxia. Strength assessment revealed lower and upper limb weakness in seven patients (25%), while two patients had weakness limited to their lower limb. P13 and P24 had normal strength in the upper and lower limbs on assessment.

Strabismus was observed in eight patients (28.6%), seven of whom also had nystagmus. Overall, nystagmus was evident in 17 patients (60.7%). In addition to nystagmus and strabismus, P5 had amblyopia and anisocoria. P26 had an absent pupillary response. Four patients (14.3%) had intellectual disability, one of whom also displayed aggressive behavior and was diagnosed with ADHD. Other neurological signs that were reported in the physical examination records include positive Babinski sign in three patients, dystonia in three patients, facial dyskinesia in one patient, tongue fasciculations in one patient, dysdiadochokinesia in one patient, and Gower sign in one patient.

Skeletal deformities, including kyphosis, scoliosis, equinovarus, and coxa valga, were observed in ten patients (35.7%). Microcephaly was evident in five patients (17.9%), while relative macrocephaly was observed in 1 patient. Dysmorphology assessment revealed some form of facial dysmorphism, including frontal bossing, prominent forehead, and depressed nasal bridge, in six patients (21.4%).

Review of systems revealed some form of gastrointestinal disease in 12 patients (42.9%), most commonly constipation (75.0% of cases) and gastroesophageal reflux disease (50% of cases).

Disturbed sleep manifesting as nighttime awakening was reported in five patients and as central and obstructive sleep apnea in three patients. Other less common signs and symptoms included emaciation, excessive crying, recurrent infections, reflex autonomic dystrophy, and urinary incontinence.

Of note, serial physical examinations were performed for most patients in the non-Saudi Arabian cohorts. As such, the following calculations apply to the 12 patients from those cohorts. The mean age at which nystagmus was noted is 34.6 months (median = 32 months, range 13–58 months), while strabismus was discovered earlier (mean = 25 months, median = 23 months, range 15–36 months). Increased appendicular tone was revealed on physical examinations at a mean age of 27 months (median = 28.6 months, range = 12–58 months), decreased axial tone at 27.2 months (median = 27 months, range = 21–36 months), and muscle weakness at 32.2 months (median = 27 months, range- 23-44 months). DTR examination revealed hyperreflexia at a mean age of 29.7 months (median = 30 months, range = 23–36 months) and hyporeflexia at a mean age of 35.5 months (median = 33 months, range = 25–57 months).

### Imaging studies

Brain magnetic resonance imaging (MRI) was performed on 26 patients (92.9%). MRI was repeated for six of those patients (23.1%). No MRI results were found in the medical records of P2 and P9. The average age at which the first MRI was performed was 3 years (mean = 36.9 months, median = 27 months, range 17 months to 8.6 years).

**Fig. 2:**
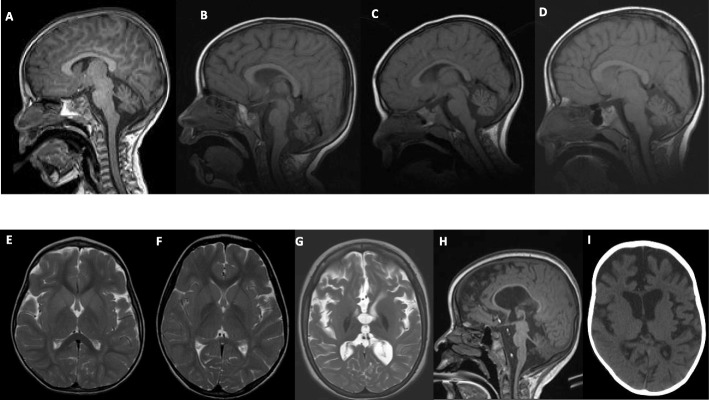
Radiographic Imaging Data for the Study Cohort. **a-d**: Representative MRI images for the affected individuals P14 (at age 3 years 2 months), P15 (at age 3 years 2 months), P19 (at age 4 years 3 months), and P20 (at age 4 years 7 months) showing cerebellar atrophy. **e**: MRI at 3 years and 2 months showing altered signal intensity in the globus pallidus of P15. **f**: MRI at 4 years and 3 months showing bilateral symmetrical subtle low T2 signals noted in the globus pallidus, substantia nigra, and dentate nuclei of P19. **g**: MRI at 6 years and 9 months showing bilateral globus pallidus low signal intensity likely representing iron deposition in P18. **h**: MRI at 6 years and 9 months showing brain, cerebellar and brainstem atrophic changes in P18. **i**: CT at 6 years showing global brain atrophy in P26

Variable degrees of cerebellar atrophy were observed in all patients except P4, P16, and P24, who had normal MRI findings at the ages of 1.75 years, 1.42 years, and 3 years, respectively. No repeated MRI were performed for those patients. Two patients had an initially normal cerebellum (P3 at 1.91 years and P17 at 3.83 years), but repeated MRI later showed cerebellar atrophy (P3 at 3 years and P17 at 7.5 years). In our cohort, cerebellar atrophy was noticed on MRI at an average age of 3.36 years (range = 1–8.6 years). In addition to cerebellar atrophy, four patients (P18, P26, P27, and P28) had diffuse cerebral atrophy with widening of cerebral sulci and thinning of corpus callosum noticed at an average age of 4.16 years (range = 3–6 years). There was an increased T2 and FLAIR signal in the periventricular, peritrigonal, and/or parieto-occipital white matter of four patients (16.6%) noted on MRI at an average age of 2.98 years (range = 2.25–4.25 years). Bilateral symmetric altered T2 signal intensities were noted within the globi pallidi of six patients (23.1%), three of whom had hypointense lesions, two had hyperintense lesions, and one was unspecified. Additionally, bilateral symmetric low signal intensity was noted in the substantia nigra and dentate nuclei of P19. Representative MRI images of some of these findings are shown in Fig. 2.

Other findings were more variable and included retro-cerebellar fluid collection (arachnoid cyst) in P3, brainstem thinning and atrophy of the pons in P7, nonspecific increased signal intensity at the dorsal aspect of the pons in P22, and atrophic dentate nuclei and symmetrical T2 hyperintense lesions of the head of the caudate nucleus and anterior horn of putamen bilaterally in P23. In addition to cerebellar atrophy, P25 had mild adenoid hypertrophy and mild mucosal sinus disease of ethmoid air cells and maxillary sinuses.

### Neurophysiologic testing

EEG recordings were performed for 18 patients (64.3%). The findings were normal in eight of those patients (44.4%), although four (P8, P14, P16, and P17) had positive seizure history. Three patients (P1, P6, and P27) had slow posterior background rhythm suggestive of bilateral diffuse cerebral dysfunction. Epileptiform discharges were observed in six patients (33.3%), all of whom had positive seizure history. No EEG recording was found for P18, despite having positive seizure history. Overall, 12 patients (42.9%) had at least one reported seizure at an average age of 39 months (range 6 months to 7.3 years). All the reported seizures were generalized, except for two patients (P5 and P16) who had partial seizures. The type of seizure was not reported for two (P15 and P18) of the 12 patients.

Electromyography (EMG) testing was performed on eight patients (28.6%) and revealed motor-more-than-sensory axonal-predominant polyneuropathy or denervation in six of those patients (75.0%). No electrophysiological evidence of axonal or demyelinating polyneuropathy, defect in the neuromuscular junction transmission, or a primary disorder of muscle was found in the remaining two patients (P3 and P20), although the EMG on one of them (P3) showed evidence of L4-L5 radiculopathy.

Nerve conduction velocity (NCV) tests were performed on nine patients (32.1%). These studies revealed distal axonal-type motor neuropathy in seven of those patients (77.8%), while two (P4 and P23) had normal findings.

Eleven patients (39.3%) were tested by flash visual evoked potentials (FVEP), eight (72.7%) of whom showed delayed FVEPs with reduced amplitude bilaterally. Interestingly, electroretinography (ERG) was done on seven of those patients and revealed normal findings in all except P12 who had intense dysfunction of the retina bilaterally compromising the rod system. P10, P11, and P19 had normal findings on FVEP testing.

Brainstem auditory evoked response (BAER) was tested in 16 patients (57.1%). Fourteen patients (87.5%) had an abnormal response signifying some degree of hearing loss. P20 and P23 had normal findings.

### Laboratory results

Aspartate aminotransferase (AST) and Alanine Aminotransferase (ALT) levels were quantified in 23 patients (82.1%). All patients had an elevated AST to ALT ratio. P9 had an elevated ALT and normal AST at one point for unknown reasons. Lactate dehydrogenase (LDH) was ordered for 10 patients (35.7%), and it was elevated in all 10.

Other labs that were ordered for some patients include CBC, serum amino acids, lactic acid, ammonia, acylcarnitine profile, urine organic acids, and CK. P28 had high serum cholesterol levels and high arginine levels. CBC revealed slightly elevated platelet count in P27 and low MCV in P25 and P24. The organic acid profile of P25 revealed decanedioic acid and low carnitine. Additionally, this patient and P22 had elevated alkaline phosphatase levels. Plasma amino acid profile of P1 showed mildly elevated glycine. Carnitine was low in P21, who also had elevated serum sodium, chloride, and urea levels. Microcytic hypochromic anemia was reported in P15. Organic acid profile of P7 showed elevated succinic acid and slightly low total carnitine.

A nerve biopsy was taken from P11 around 36 months of age and showed few but very prominent axonal swellings. Intra-axonal inclusions and abnormal mitochondrial morphology were seen in the nerve biopsy of P14. His muscle biopsy showed neurogenic atrophy with intra-sarcoplasmic glycogen. A muscle biopsy from P15 revealed mild to moderate fiber size variation with scattered atrophic change, sub-sarcolemmal glycogen accumulation in multiple fibers, and occasional dark blue fibers on NADH staining and scattered subsarcolemmal mitochondrial accumulation. Neuropathic changes were observed on the muscle biopsy taken from P18.

### Molecular genetic testing

Sequencing of *PLA2G6* revealed 11 different homozygous variants and 10 different compound heterozygous pairs for a total of 16 unique individual variants, eight of which were not previously reported in literature. The variants are summarized in Table 1.

**Table 1:** Summary of Mutations Data for the Study Cohort

Family ID	Patient ID	Variant	Homozygous	Compound Heterozygous	HGMD Reference
F1	P1	NM_003560.2: c.2370_2371del (p.Tyr790*); c.1506G > C (p.Lys502Asn)	N	Y	CM063050; CM063018
F1	P2	NM_003560.2: c.2370_2371del (p.Tyr790*); c.1506G > C (p.Lys502Asn)	N	Y	CM063050; CM063018
F2	P3	NM_003560.2: c.2370 T > G(p.Y790*); deletion of exon 2	N	Y	CM063050; This study
F3	P4	NM_003560.2: c.1982C > T (p.Thr661Met); c.109C > T (p.Arg37*)	N	Y	CM138339; CM063024
F4	P5	NM_003560.2: c.1613G > A (p.R538H); c.319dupC (p.L107PfsX10)	N	Y	CM165308; This study
F5	P6	NM_003560.2: c.1427 + 1G > A; c.1539del (p.Trp514fs)	N	Y	CS090239; This study
F6	P7	NM_003560.2: c.1613G > A (p.R538H); c.2370 T > G (p.Y790X)	N	Y	CM165308; CM063050
F7	P8	NM_003560.2: c.1903C > T (p.R635X); c.1798C > T (p.R600W)	N	Y	CM063025; CM145848
F8	P9	NM_003560.2: c.404 T > C (p.F135S); deletion of exon 6	N	Y	CM063051; This study
F9	P10	NM_003560.3: c.2370 T > G(p.Y790*); c.1046A > C(p.H349P)	N	Y	CM063050; This study
F10	P11	NM_003560.2: c.1997C > T (p.Thr666Ile)	Y	N	This study
F11	P12	NM_003560.2: c.2078 T > C (p.Leu693Pro); c.(−46 + 1_-1)_(209 + 1_210–1)	N	Y	This study; This study
F12	P13	NM_003560.2: c.1771C > T (p.R591W)	Y	N	CM090233
F13	P14	NM_003560.2: c.1125delA (p.Val376Trpfs*14)	Y	N	CD1310226
F14	P15	NM_003560.2: c.1911delC (p.Ser637Argfs*29)	Y	N	CD1310227
F14	P16	NM_003560.2: c.1911delC (p.Ser637Argfs*29)	Y	N	CD1310227
F15	P17	NM_003560.2: c.2218G > A (p.G740R)	Y	N	CM1310225
F16	P18	NM_003560.2: c.2070_2072delTGT (p.Val691del)	Y	N	CD063595
F17	P19	NM_003560.2: c.1772G > A (p.R591Q)	Y	N	CM063032
F18	P20	NM_003560.2: c.2251G > A (p.E751K)	Y	N	CM063028
F19	P21	NM_003560.2: c.2070_2072delTGT (p.Val691del)	Y	N	CD063595
F20	P22	NM_003560.2 c.1933C > T (p.R645*)	Y	N	CM121238
F15	P23	NM_003560.2: c.2218G > A (p.G740R)	Y	N	CM1310225
F19	P24	NM_003560.2: c.2070_2072delTGT (p.Val691del)	Y	N	CD063595
F21	P25	NM_003560.2: c.1933C > T (p.R645*)	Y	N	CM121238
F22	P26	NM_003560.2: c.1125delA (p.Val376Trpfs*14)	Y	N	CD1310226
F23	P27	NM_003560.2: c.1933C > T (p.R645*)	Y	N	CM121238
F19	P28	NM_003560.2: c.2070_2072delTGT (p.Val691del)	Y	N	CD063595

*Abbreviations*: *N* no, *Y* yes, *HGMD* human gene mutation database

All variants for patients in this cohort were initially tabulated and grouped according to the underlying variant type such as truncating or non-truncating (missense and in-frame deletion). Averages for age at initial onset of overall symptoms as well as at initial loss of skills in specific developmental categories were calculated for the entire cohort and for each underlying variant-type. These averages were then graphed to evaluate for potential correlations between underlying variant-type and phenotype.

The variants for all patients in this cohort were subsequently analyzed and common variants were grouped together. Graphs (Supplemental Graphs S1-S5) were then created to reflect the age of onset of initial concern or onset of specific phenotype (i.e. age at loss of skills in specific developmental category) for each variant identified within the cohort. Some patients were plotted twice, if they were compound heterozygous for variants in *PLA2G6,* to ensure that the potential impact of each variant on phenotype was considered in our analysis. Given that this is a retrospective study, data was not available for every patient in every category. In those cases, nothing was plotted for the respective patients which resulted in a variable number of datapoints plotted on each graph. In addition, if retrospective chart review clearly confirmed that skills within a specific developmental domain were never achieved at all, this was designated as 0 months for age of onset of initial loss of skills in that domain.

**Fig. 3:**
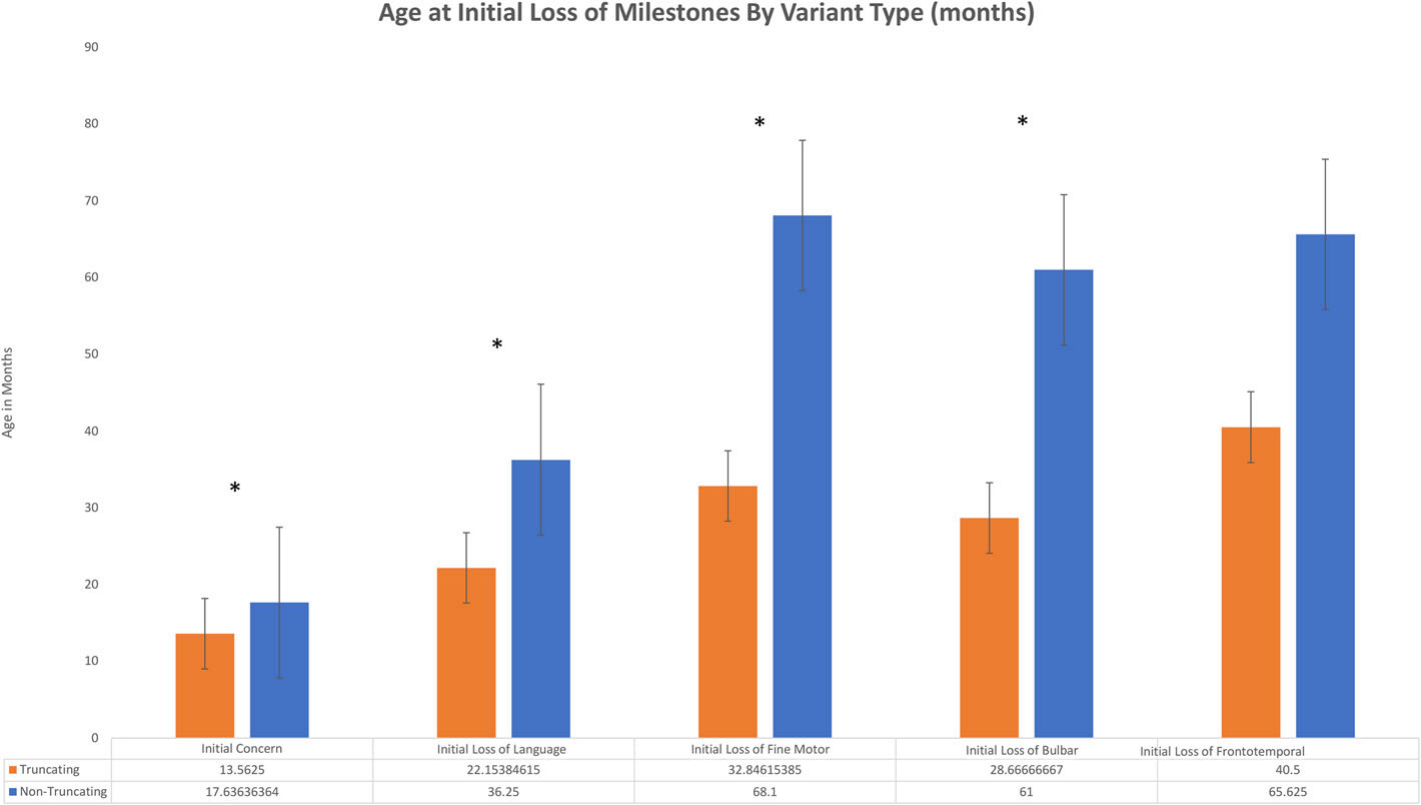
Age at Initial Loss of Milestones By Variant Type With Standard Errors (*p value <0.05)

Specifically, analysis of our data by underlying type of variant revealed that patients with truncating variants for one or both of their underlying variants had an earlier average age at the time of initial concern and an earlier average onset of initial regression across all developmental domains. This was true when compared to the averages across the entire cohort and when compared to the combined averages for non-truncating variants. This is not entirely unanticipated since truncating variants are typically more deleterious to downstream enzyme function than other variations and thus this was our expected hypothesis. Using a one-tailed T-test we were able to show there was a statistically significant difference when comparing truncating variants compared with non-truncating in the time of initial concern (*p* = 0.04), initial loss of language (*p* = 0.001), initial loss of fine motor skills (*p* = 0.009), and initial loss of bulbar skills (*p* = 0.007) (Fig. 3). We did not observe a statistically significant difference in initial loss of temporal skills (*p* = 0.11) but note the sample size in both cohorts was smaller (*n* = 8) and thus not adequately powered. The average age for initial concerns to be noted for the entire cohort was 15 months, while for truncating variants it was 13.5 months (*n* = 16, standard deviation (SD) 3.9 months), for non-truncating variants it was 17.5 months (*n* = 11, SD 7.7 months). This is consistent with the relatively well-established average age of onset of INAD symptoms between 6 months and 3 years of age.

Mean initial loss of language for truncating variants was 22.1 months (*n* = 13, SD = 7.2) and non-truncating variants was 36.3 (*n* = 8, SD = 12.2). Mean initial loss of fine motor skills for truncating variants was 32.8 months (n = 13, SD =17.7) and non-truncating variants was 68.1 months (*n* = 10, SD = 45.4). Mean initial loss of bulbar skills for truncating variants was 28.7 months (*n* = 12, SD = 14.6) and non-truncating variants was 61.0 (n = 10, SD = 38.01. Mean initial loss of temporal skills for truncating variants was 40.5 (n = 8, SD = 14.8) and non-truncating variants was 65.6, (n = 8, SD = 53.4).

## Discussion

In our cohort, loss of balance was the most commonly reported initial concern, followed by developmental delay. The average age at which initial concerns arose was 15 months. Following the initial symptoms, the disease progressed relentlessly, leading to speech and gross motor regression that predominated early in the disease, followed by later deterioration in fine motor and bulbar functions. Temporo-frontal signs were among the last milestones to be lost. The mean age of death in our study was 9.9 years with respiratory causes predominating, likely secondary to bulbar dysfunction. This pattern of disease progression is consistent with previous reports [5–10, 18].

Our INAD cohort experienced early truncal hypotonia and appendicular hypertonia followed by spastic tetraparesis, usually with hyperreflexia in the early stages with progression to hyporeflexia or areflexia later in the disease course. Strabismus and nystagmus are common and early features of the disease [8]. Serial physical examinations performed on our cohort revealed a similar pattern.

The prenatal and postnatal history of INAD has rarely been reported in literature [19]. In our study, three patients (10.7%) experienced an episode of hypoxia postnatally that necessitated an admission to the NICU. Two other patients were admitted to the NICU, one for jaundice and respiratory distress and the other for thrombocytopenia. Although postnatal complications requiring admission to NICU did not seem to affect the clinical features or time of onset of INAD, they seemed to occur at a higher frequency compared to the general population [20, 21].

Seizures in INAD patients may develop early or late in the disease course and are reported to occur in a minority of patients [5, 7, 9–11]. In our cohort, 12 patients (42.9%) had at least one reported seizure at an average age of 39 months. All the reported seizures were generalized onset, except in two who had either a focal onset seizure localizing to the temporal lobe or focal to bilateral tonic clonic seizures. The presence of seizures in almost half the patients in our cohort and the wide range of ages at which the seizures develop should encourage clinicians to screen their patients for seizures more frequently and at different timepoints.

Neurophysiologic tests commonly reveal delayed signals with reduced amplitudes on flash visual evoked potential (FVEP) test, distal axonal-type sensorimotor neuropathy on nerve conduction velocity (NCV) test, evidence of denervation on electromyography (EMG), delayed responses on brainstem auditory evoked response (BAER) test, and fast rhythms on electroencephalogram (EEG) [8, 19]. These findings are consistent with our results, as most tested patients had bilaterally delayed FVEPs with reduced amplitudes, motor-more-than-sensory axonal-predominant polyneuropathy or denervation on EMG, distal axonal-type motor neuropathy on NCV, and an abnormal BAER signifying some degree of hearing loss. Three patients had a slow posterior background rhythm suggestive of bilateral diffuse cerebral dysfunction and 6 had epileptiform discharges on EEG.

Two patients manifested psychiatric symptoms, including excessive crying, ADHD and aggressive behavior. Salih et al. (2013) [19] reported two INAD patients with psychiatric symptoms and postulated that the pathological involvement of locus ceruleus, which has been documented in patients with *PLA2G6* pathogenic variants [5]., may explain these manifestations.

Autonomic involvement in INAD may present as constipation or urinary incontinence [8, 22]. Indeed, nine of our patients were reported to have constipation requiring treatment, and 1 had urinary incontinence.

Initial brain MRI performed on 26 of our patients revealed cerebellar atrophy as a consistent feature observed in all except 5 patients. While three of those patients did not receive any additional neuroimaging tests, it is not inappropriate to assume that cerebellar atrophy would have developed at a later age [23]. Indeed, the two other patients who did receive additional neuroimaging at a later age had cerebellar atrophy. These findings reveal that although cerebellar atrophy is a universal feature of INAD, consistent with previous observations [5, 7, 10, 11], it may be completely absent at the time of presentation. Clinicians, therefore, should be careful to avoid ruling out INAD based on initial neuroimaging findings and should consider follow-up imaging studies at a later age and particularly at the time of diagnosis. Other MRI findings that we observed in our cohort, including diffuse cerebral atrophy and abnormal signals in periventricular white matter, have been occasionally described in previously published INAD cases [7, 23]. Generalized cerebral atrophy was also seen as widening of cerebral sulci and thinning of corpus callosum among four patients in our cohort. We also observed increased T2 and FLAIR signal in the periventricular, peritrigonal, and/or parieto-occipital white matter in four patients. Globus pallidus hypointense lesions representing iron deposition appeared in a repeat MRI done later in the disease course of one patient. This is consistent with the findings in literature, which indicate that iron accumulation seems to become more appreciable later in the disease course [9]. Bilateral hyperintense lesions of the head of the caudate nucleus and anterior horn of putamen, peculiar findings found in a few reports [5, 6, 24], were also observed in one of our patients.

Screening bloodwork can also be a powerful tool, providing clues about the potential diagnosis of INAD. It has been previously reported in the literature that AST and LDH elevations are consistent in patients with INAD [25]. A similar pattern is not reliably reproduced among patients with other conditions in the *PLA2G6*-disease spectrum and thus it has been proposed that AST and LDH elevations are relatively specific for *PLA2G6*-related INAD. While the LDH elevations can be reasonably presumed to reflect generalized tissue damage, the AST elevations may be related to the role of mitochondrial AST as a plasma membrane protein involved in free fatty acid uptake. As such, it has been postulated that mitochondrial AST is released from the membrane in the setting of damage and disturbed metabolic processes [26]. The findings in the international cohort in this study were consistent with the aforementioned, published patterns. Specifically, 91% of tested patients in the present study had AST elevations. Similarly, LDH was elevated in 100% of those tested in our cohort. Of note, one of the patients with initially elevated AST and normal ALT levels subsequently had normal AST and elevated ALT for unknown reasons. Although this latter result could reflect mild viral illness, it is not uncommon to note an increase in both AST and ALT in that setting.

Once a molecular diagnosis is established, healthcare professionals and families often wonder whether that specific variant has been reported in any patients previously and if so, how often and what sort of genotype-phenotype correlations have been established. Because INAD is ultra-rare, our sample size is somewhat limited. In addition, because our data collection was entirely retrospective, age specific datapoints may not be as precise as they would be for a prospective study. Regardless, there were some interesting correlations and trends that are worthy of mention.

The greatest variability between patients with different types of underlying variants occurred for average age at initial loss of fine motor, bulbar and frontotemporal skills. It could be argued that because these developmental areas are, on average, affected later in the course of the disease, perhaps there was less consistent documentation of developmental milestone losses during that time because the medical care of these patients had become increasingly complex. That could certainly lead to greater variability in the time frame at which specific developmental losses were noted, if at all. Despite the overall greater variability, there is a trend for later onset of symptoms in all three of these domains (fine motor, bulbar and frontotemporal) among patients with one or two underlying non-truncating variants, when compared to patients with underlying truncating variants.

When analyzed on a smaller scale to assess possible specific genotype-phenotype correlations, the results were not as revealing. This is partly because of the small number of cases, but also because a single Saudi founder variant was observed in 18% of cases. Also of note, there were two variants, p.R645* and p.S637Rfs*29, that consistently plotted in the lowest 50% of datapoints on the x-axis across four of the five developmental phenotype graphs. Variant p.S637Rfs*29 also plotted in the lowest 50% for age at initial loss of language, but the p.R645* variant did not. This correlates with a consistently earlier onset of overall symptoms; earlier than at least half of the rest of the cohort. Though, further correlations cannot be drawn since the relative ranking of these two variants within that earliest 50% is quite variable across the different developmental domains analyzed. Among previously published manuscripts on INAD, genotype-phenotype correlations were not identified or established except among one cohort described in a 2013 paper [19]. Similar to the findings in the present cohort, the authors of that 2013 paper noted a correlation between earlier onset of symptoms and progression of functional disability with specific types of underlying variants, notably among variants predicted to be the most damaging (mostly nonsense or frameshift). However, the general variability in phenotype even among patients that share the same genotype or that are within the same family, as seen in the present study and in previously published cohorts, suggests that there may be unidentified environmental or additional genetic factors contributing to the phenotype.

We report 16 unique individual *PLA2G6* pathogenic variants (NM_003560.2), 8 of which are novel variants that were not found in the Human Gene Mutation Database (HGMD). The novel *PLA2G6* variants include 7 found in compound heterozygous state: deletion of exon 2 in P3, c.319dupC (p.L107PfsX10) in P5, c.1539del (p.Trp514fs) in P6, deletion of exon 6 in P9, c.1046A > C(p.H349P) in P10, and c.2078 T > C (p.Leu693Pro); c.(− 46 + 1_-1)_(209 + 1_210–1) in P12. A novel homozygous missense variant was also reported in P11 (NM_003560.2: c.1997C > T (p.Thr666Ile)).

Finally, we note an absence of observed differences across the Saudi, American, and European populations and posit no change in severity based on ethnic background. The strongest correlation to disease severity observed was the presence of a truncating variant.

## Conclusions

In conclusion, we report the phenotypic and genetic findings in 28 INAD patients, the largest number reported in any single study to date. This study defines the natural history of INAD and reports eight novel *PLA2G6* pathogenic variants. We documented the pattern of neuroregression and noted a number of common systemic findings, typical brain imaging abnormalities and laboratory findings. We observe that INAD progresses relentlessly, with no observed case of recovery of a milestone once lost. Due to the retrospective nature of our natural history study, we were limited in reporting age-specific datapoints for each clinical finding. Until a prospective natural history study of INAD that can resolve this limitation is published, we hope that this study serves as comprehensive resource for healthcare providers and families in developing a targeted care plan with appropriate surveillance, support, and education.

## Supplementary information

**Additional file 1.**

**Additional file 2.**

## Abbreviations

ACMG: American College of Medical Genetics & Genomics
INAD: Infantile neuroaxonal dystrophy
PLAN: *PLA2G6*-associated neurodegeneration
KFSHRC: King Faisal Specialist Hospital and Research Centre, Riyadh
EMG: Electromyography
NCV: Nerve conduction velocity
VEP: Visual evoked potentials
BAER: Brainstem auditory evoked response
MRI: Magnetic resonance imaging
AST: Aspartate aminotransferase
ALT: Alanine aminotransferase
CBC: Complete blood count
LDH: Lactate dehydrogenase
CK: Creatine kinase
NICU: Neonatal intensive care unit
FVEP: Flash visual evoked potential
