# Supplementary material for: The natural history of infantile neuroaxonal dystrophy
Source: Orphanet J Rare Dis. 2020 May 1;15:109. doi: 10.1186/s13023-020-01355-2 (PMC7193406; doi:10.1186/s13023-020-01355-2)
Supplement: Supplementary file 2 — Additional file 2. [file 13023_2020_1355_MOESM2_ESM.pptx]

## Slide 1
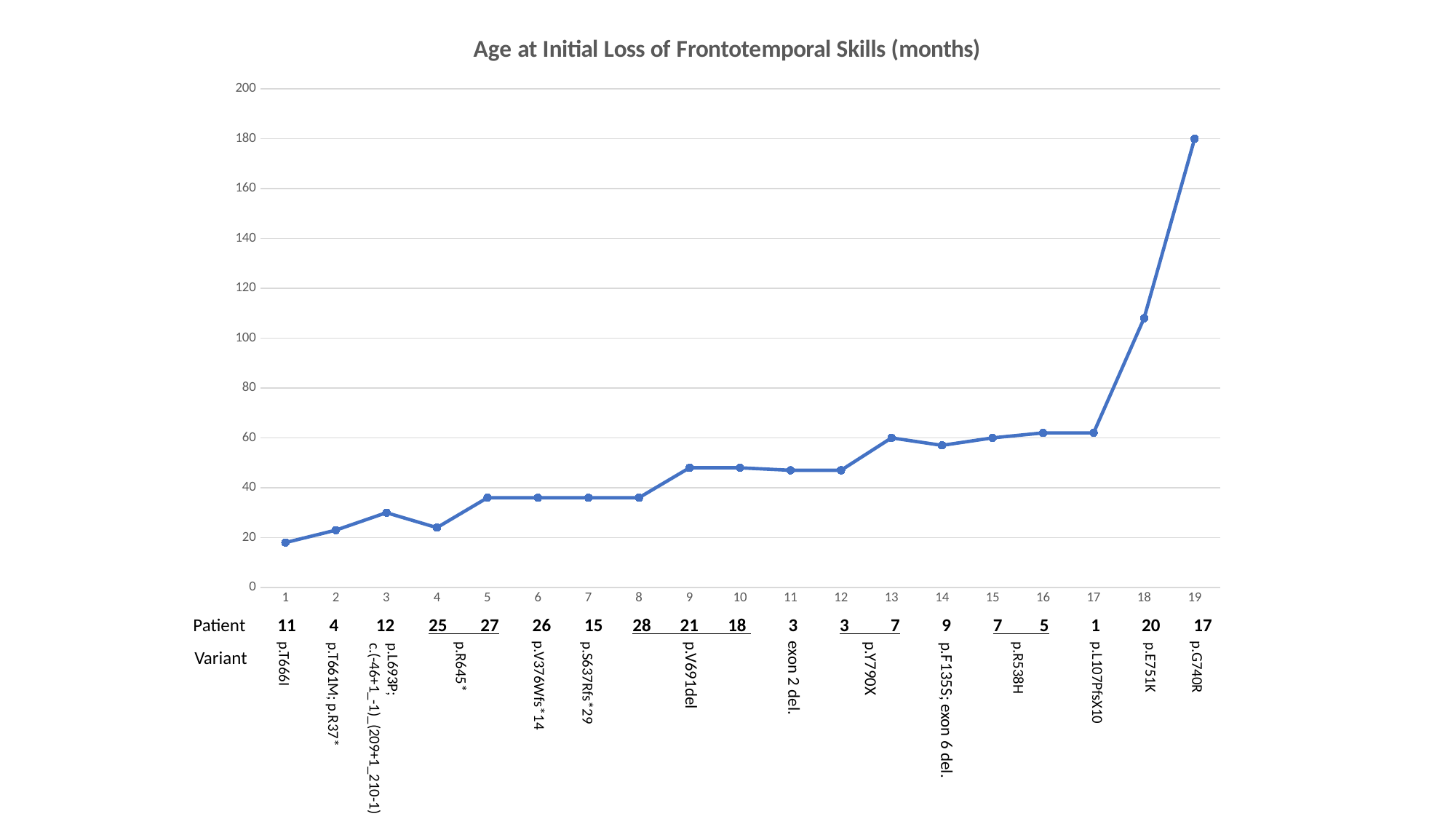

### Chart: Age at Initial Loss of Frontotemporal Skills (months)
| Category | Initial loss of frontotemporal |
|---|---|Patient
 11 4 12 25 27 26 15 28 21 18 3 3 7 9 7 5 1 20 17
Variant
p.T666I
p.R645*
p.G740R
p.E751K
p.Y790X
p.R538H
p.V691del
exon 2 del.
p.S637Rfs*29
p.L107PfsX10
p.V376Wfs*14
p.T661M; p.R37*
p.F135S; exon 6 del.
p.L693P;
c.(-46+1_-1)_(209+1_210-1)

## Slide 2
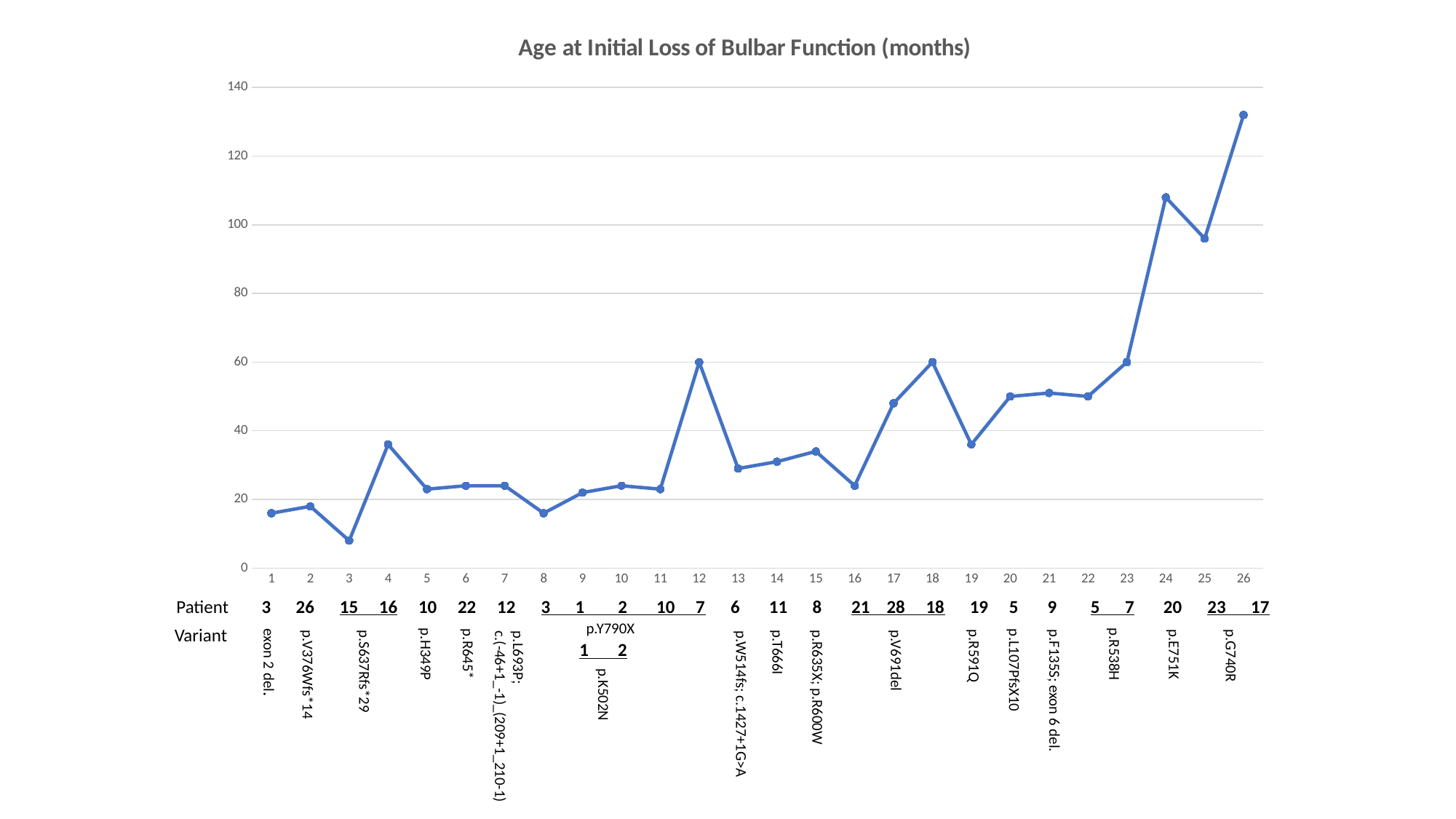

### Chart: Age at Initial Loss of Bulbar Function (months)
| Category | Initial Loss of Bulbar |
|---|---| 3 26 15 16 10 22 12 3 1 2 10 7 6 11 8 21 28 18 19 5 9 5 7 20 23 17
 1 2
Patient
p.Y790X
Variant
p.T666I
p.E751K
p.R538H
p.R645*
p.H349P
p.G740R
p.R591Q
p.V691del
exon 2 del.
p.L107PfsX10
p.S637Rfs*29
p.V376Wfs*14
p.R635X; p.R600W
p.F135S; exon 6 del.
p.K502N
p.W514fs; c.1427+1G>A
p.L693P;
c.(-46+1_-1)_(209+1_210-1)

## Slide 3
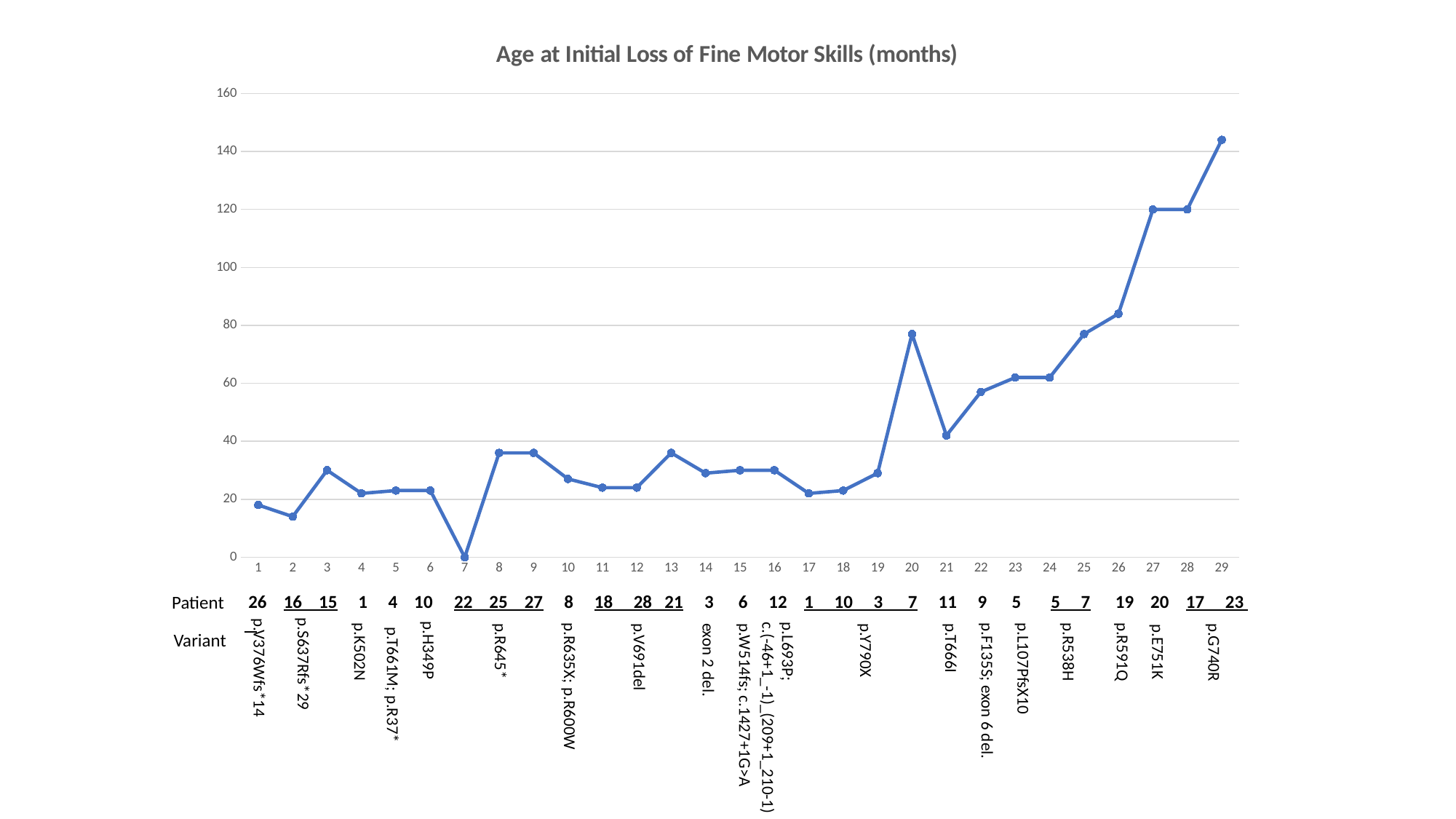

### Chart: Age at Initial Loss of Fine Motor Skills (months)
| Category | Initial Loss of Fine Motor |
|---|---| 26 16 15 1 4 10 22 25 27 8 18 28 21 3 6 12 1 10 3 7 11 9 5 5 7 19 20 17 23
Patient
Variant
p.T666I
p.H349P
p.Y790X
p.R645*
p.K502N
p.R538H
p.E751K
p.R591Q
p.G740R
p.V691del
exon 2 del.
p.S637Rfs*29
p.V376Wfs*14
p.L107PfsX10
p.T661M; p.R37*
p.R635X; p.R600W
p.F135S; exon 6 del.
p.W514fs; c.1427+1G>A
p.L693P;
c.(-46+1_-1)_(209+1_210-1)

## Slide 4
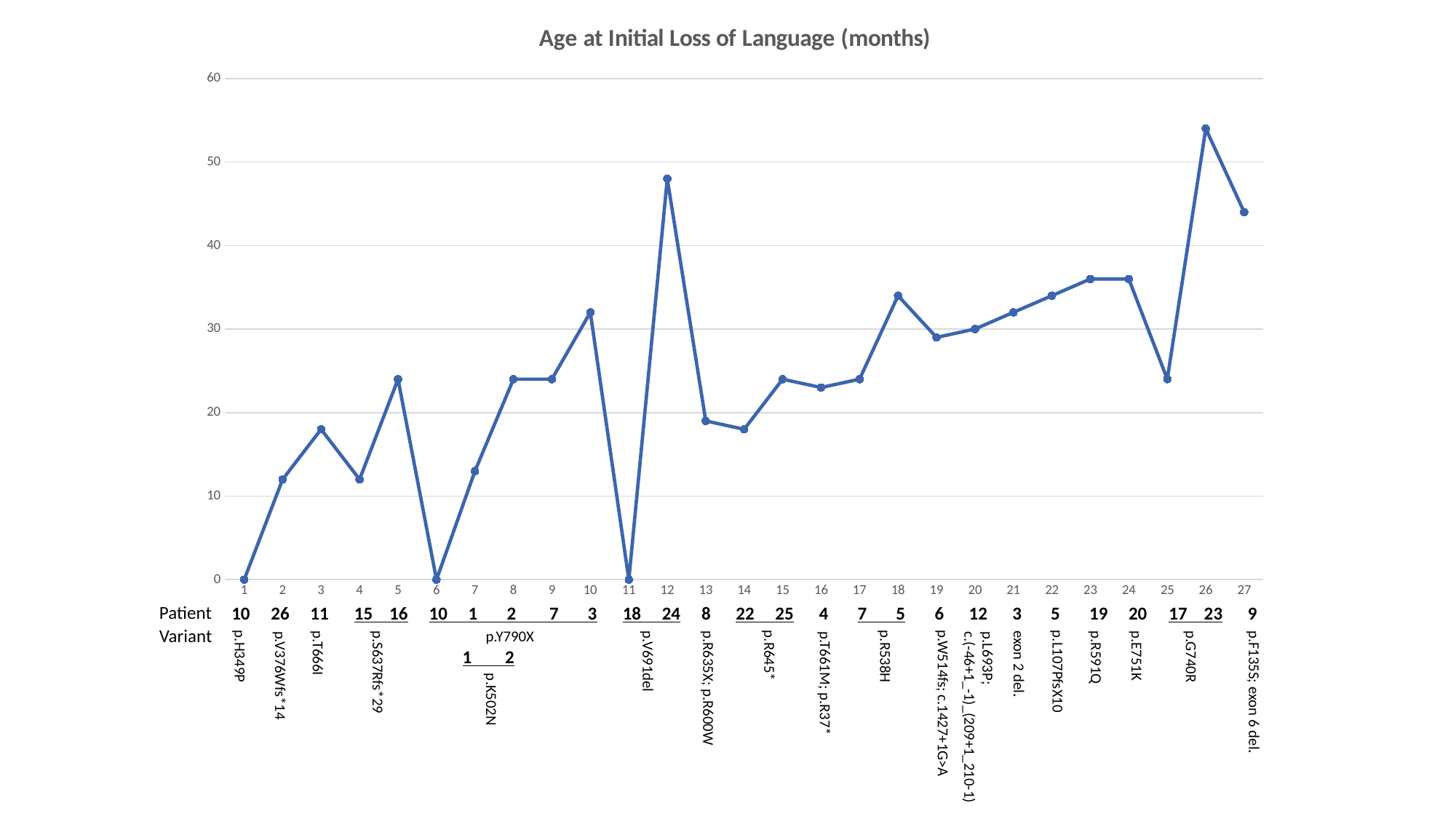

### Chart: Age at Initial Loss of Language (months)
| Category | Initial Loss of Language |
|---|---|Patient
 10 26 11 15 16 10 1 2 7 3 18 24 8 22 25 4 7 5 6 12 3 5 19 20 17 23 9
 		 1 2
Variant
p.Y790X
p.T666I
p.R645*
p.E751K
p.H349P
p.G740R
p.R538H
p.R591Q
p.V691del
exon 2 del.
p.L107PfsX10
p.S637Rfs*29
p.V376Wfs*14
p.T661M; p.R37*
p.R635X; p.R600W
p.F135S; exon 6 del.
p.K502N
p.W514fs; c.1427+1G>A
p.L693P;
c.(-46+1_-1)_(209+1_210-1)

## Slide 5
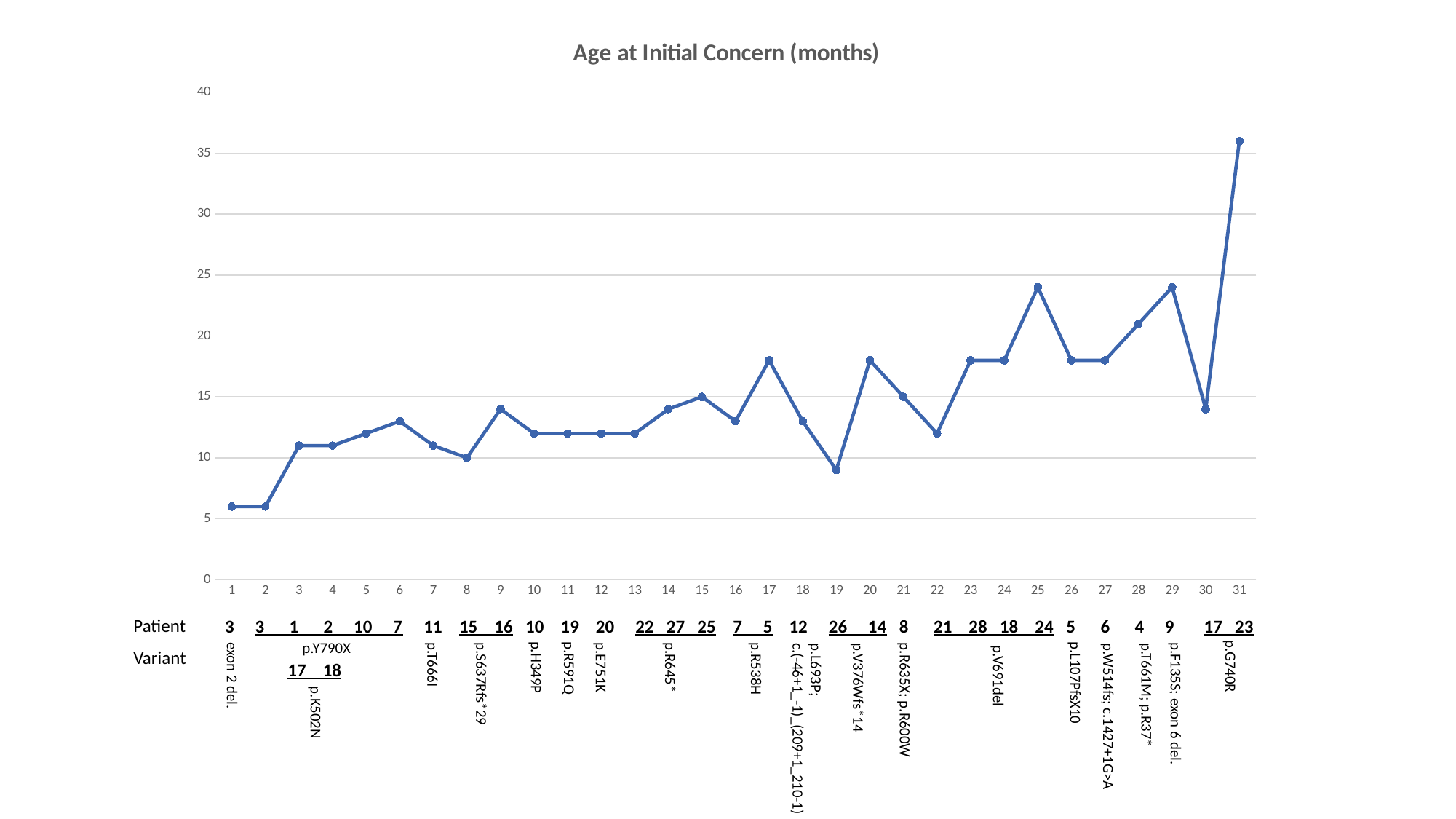

### Chart: Age at Initial Concern (months)
| Category | Initial Concern |
|---|---|Patient
 3 3 1 2 10 7 11 15 16 10 19 20 22 27 25 7 5 12 26 14 8 21 28 18 24 5 6 4 9 17 23
 17 18
p.Y790X
Variant
p.T666I
p.R645*
p.E751K
p.H349P
p.R591Q
p.R538H
p.G740R
exon 2 del.
p.V691del
p.L107PfsX10
p.S637Rfs*29
p.V376Wfs*14
p.T661M; p.R37*
p.R635X; p.R600W
p.F135S; exon 6 del.
p.K502N
p.W514fs; c.1427+1G>A
p.L693P;
c.(-46+1_-1)_(209+1_210-1)
